# Trial-to-trial modulation of task-order switch costs survive long inter-trial intervals

**DOI:** 10.1186/s40359-022-00784-x

**Published:** 2022-03-22

**Authors:** Tilo Strobach, Mike Wendt

**Affiliations:** Department of Psychology, Medical School Hamburg, Am Kaiserkai 1, 20457 Hamburg, Germany

**Keywords:** Dual tasks, PRP, Task order, Task-order control, Executive functions

## Abstract

**Background:**

Dual-tasking procedures often involve the successive presentation of two different stimuli, requiring participants to execute two tasks in a particular order. Performance in both tasks suffers if the order of the tasks is reversed (i.e., switched) compared to the directly preceding trial. This task-order switch cost is reduced, however, if the preceding trial itself involved a task-order switch compared to a task-order repetition (Strobach in Acta Psychol 217:103328, 2021). Theoretical accounts range from assumptions of top-down implementation of a task-order control set, or passive persistence thereof, to priming based on episodic binding of tasks and temporal positions. Here, we tested these accounts by investigating whether the sequential modulation decays as a function of the inter-trial interval.

**Methods and results:**

Task-order switch costs were reliably reduced after a task-order switch (compared to after a task-order repetition) and this reduction did not decrease over inter-trial intervals ranging from 350 ms to 1,400 ms. Also replicating previous findings, for reaction times the reduction was driven by selective slowing in task-order repeat trials, suggesting increased response caution.

**Conclusions:**

Our results are consistent with preparatory processes of task-order control or with episodic integration of task-order information but argue against accounts assuming short-lived, decaying task-order sets.

## Introduction

From our real-world experience, we know that performing two simultaneous tasks in dual tasks can result in performance impairments in one or both of these tasks. In particular, there are dual-tasks costs that are realized in increased errors and/ or in an increased time to perform the tasks, as compared to their isolated performance in single tasks. Studies in the context of the paradigm of the ‘Psychological refractory period’ (PRP) investigated these costs with the presentation of two stimuli with a variable interval between the stimulus onsets (stimulus onset asynchrony; SOA), on which participants have to make choice reaction time (RT) responses. The typical finding in the PRP paradigm is that RTs of the second stimulus (RT2) increases with decreasing SOA [2–4]; this RT2 pattern is called the ‘PRP effect’.

### Sequential effects in dual tasking

Although previous research mainly aimed at identifying the origin of the PRP effect (often attributing it to a hard-wired or strategic processing bottleneck located at the stage of response selection, see [2, 5], for overviews), recent studies have emphasized questions of task coordination and order control under conditions of rapid successive administration of stimuli for different tasks. Among others, this research demonstrated that changing the order of task presentation from the preceding trial N − 1 to the current trial N (i.e., different task orders and therefore a task-order switch) yields a performance impairment for both tasks in comparison to the same task order and therefore a task-order repetition across these trials (henceforth task-order switch costs, e.g., [6]. Task-order switch costs have been observed even when regular sequences of order repeat and order switch trials provided foreknowledge concerning the task order of the upcoming trial (e.g., [7, 8]), suggesting a processing limitation that cannot easily be overcome by anticipation/preparation. Intriguingly, however, task-order switch costs are modulated by the “order sequence” status of the predecessor trial.

Specifically, Strobach, Kübler, and Schubert [1] demonstrated reduced task-order switch costs when trial N − 1 itself involved a reversal of task order (i.e., the task order changed from the penultimate trial N − 2 to trial N − 1) compared to when this trial N − 1 involved a repetition (i.e., the task order did not change from trial N − 2 to trial N − 1). This reduction occurred for both tasks of the trial, affected RTs and error rates, was observed for two-choice tasks and three-choice tasks, as well as for relatively short and relatively long SOAs.

### Mechanisms of sequential effects in dual tasking

Various theoretical accounts seem worth being considered concerning the mechanisms underlying the reduction of the task order switch costs (see also discussion in 1). The first account is inspired by current models of conflict adaptation, in which the occurrence of distractor-evoked conflict is assumed to trigger the recruitment of additional “cognitive control” or attentional adjustment, allowing the system to deal more efficiently with future conflict situations [9, 10] (for a current overview, see [11]). According to this account, one might assume that the detection of task order mismatch on consecutive trials results in the adoption of some task-order control processes which prepare the system for another order switch, thus reducing the cost of switching. Note that, viewed from this perspective, different processes may underlie the occurrence of task-order switch costs and the reduction thereof after an order switch trial (i.e., recruitment of control processes for an increased activation of task-order sets, [12]). A different perspective can be taken, however, giving rise to an account that assumes accumulation of task-order priming across multiple trials. This becomes most obvious, if trials are categorized according to the individual order of tasks in trial N and trial N − 1 rather than according to task-order sequence in trial N and trial N − 1. More precisely, letting *AB* represent a trial in which task A is presented before task B, the four conditions examined when analyzing task-order switch costs after task order repetitions vs. switches translate into the sequences of trials depicted in Table 1.

**Table 1:** Relationship of task order sequence and task-order in successive trials N − 2, N − 1, and N for current trials involving task order AB

Previous trial N − 2	Previous trial N − 1	Current trial N	Task-order sequence
AB	AB	AB	Same order → same order
BA	AB	AB	Different order → same order
AB	BA	AB	Different order → different order
BA	BA	AB	Same order → different order

As can be discerned from Table 1, assuming any kind of task-order priming that has the effect of facilitating responding in trials with a particular task-order AB more strongly the more frequently and the more recently AB occurred in the history of trials would predict a ranking of performance for the four trial types in the order listed in the table (i.e., best performance for the condition depicted in the top line, worst performance for the conditions depicted in the bottom line, etc.). Overall, the performance pattern observed by Strobach et al. [1] closely matched this ranking, albeit RTs of trials associated with an order switch in both trial N and trial N − 1 (e.g., AB → BA → AB) did not differ in accord with the predicted scheme from RTs of trials associated with an order-switch in trial N and an order repetition in trial N − 1 (e.g., BA → BA → AB). (We will address this discrepancy between the RT and error result in more detail in the Discussion.) The trial-to-trial modulation of task order switch costs thus seems consistent with accounts assuming an accumulation of the outcome of the very same process by which task-order switch costs are brought about in the first place, weighted according to the recency and frequency of its occurrence. Put differently, executing tasks in a particular order may induce a mental set favoring the same task order on the following trial and this bias is strengthened by further application of the same task-order.

Although, at the current stage, possible explanations for the sequential modulation of task-order switch costs do not appear sufficiently elaborated to allow deriving detailed predictions, accounts of top-down preparation for an upcoming trial vs. persisting activation of previous applications of task-order are likely to differ with regard to their predictions concerning the consequences of varying the length of the inter-trial interval. Whereas the former “strategic” measures would be expected to rely on the implementation of a task-order set in working memory and maintenance thereof as long as it is “deemed useful”, the latter—lacking such maintenance—would be expected to be subject to some form of decay after application. The former “strategic” account” would thus predict that the sequential effect of task-order switch costs increases over short inter-trial intervals and remains unaffected during longer intervals, whereas the latter decay account would assume a maximum effect at short inter-trial intervals and a steady reduction with increasing interval length. Interestingly, evidence for set decay was observed in an investigation of the Gratton effect (i.e., reduction of the distractor congruence effect after incongruent compared to after congruent trials N − 1). Specifically, administering a face-word conflict task, Egner et al. [13] found that the Gratton effect continuously diminished when the inter-trial interval increased. The authors interpreted these results to indicate a “’phasic’ version of conflict adaptation, where processing conflicts elicit a short-lived reinforcement of top-down attentional set “ (p. 7), refuting “strategic” accounts based on expectation of the upcoming congruency level.

Leaving aside such top-down processes, however, a simple way to account for the sequential modulation of task-order switch costs is to assume incidental bindings of task (identity) and temporal position within a trial in episodic memory. More precisely, this view would hold that responding to a task stimulus, presented in the first or second temporal position of stimulus occurrence in the trial tends to retrieve memory episodes of trials presented earlier in the sequence, containing the same task or the same temporal position. Responding is then interfered by mismatches between current task-temporal position conjunctions and conjunctions contained in the retrieved episodes (i.e., partial mismatches) compared to matching episodes containing the same task-position conjunction. Although such an account makes no reference to top-down or “strategic” preparation, there is no obvious reason to assume a decrease in its effectiveness when the time interval between trials is increased, as long as the assumed bindings are sufficiently stable. Accordingly, previous studies investigating bindings of (low-level) stimulus and response features observed effects that lasted for several seconds of inter-trial interval, without any significant reduction (e.g., [14]). Similar to top-down preparation, accounting for the sequential modulation of task-order switch costs in terms of episodic retrieval would therefore appear consistent with a lack of a reduction of the effect across inter-trial intervals.

### The present study

In this study, we investigated task-order switch costs and their trial-to-trial modulation under conditions of three inter-trial intervals of different lengths. As laid out above, to some extent, different expectations can be derived from the different accounts. Top-down (strategic) preparation would seem likely to be associated with an increase of the sequential effect on task-order switch costs from short inter-trial intervals to an inter-trial interval of medium size, which allows for full implementation of the strategic set (i.e., a more pronounced effect of the sequential modulation for a medium interval). Once implemented, however, this set should be actively maintained to affect impending stimulus processing accordingly, hence we predict absence of decay when comparing the medium-sized to the long inter-trial interval. By contrast, accounting for the sequential modulation in terms of a passively persisting, “phasic” state brought about by executing tasks in a particular order in the previous trial would predict a steady decay function, that is, monotonic reduction of the effect of the sequential modulation with increasing length of the inter-trial interval. Finally, assuming episodic retrieval of previous trials’ processing episodes (from long-term memory) would offer no reason for expecting any effect of the length of the inter-trial interval. We would like to stress that independently of the plausibility of these individual predictions, comparing determinants of the sequential modulation of task-order switch costs and the Gratton effect [15], such as their susceptibility to temporal aspects of stimulus presentation, seems a valuable endeavor, given the structural similarity of the two phenomena.

## Results

### *RTs (Fig. **1**)*

**Fig. 1:**
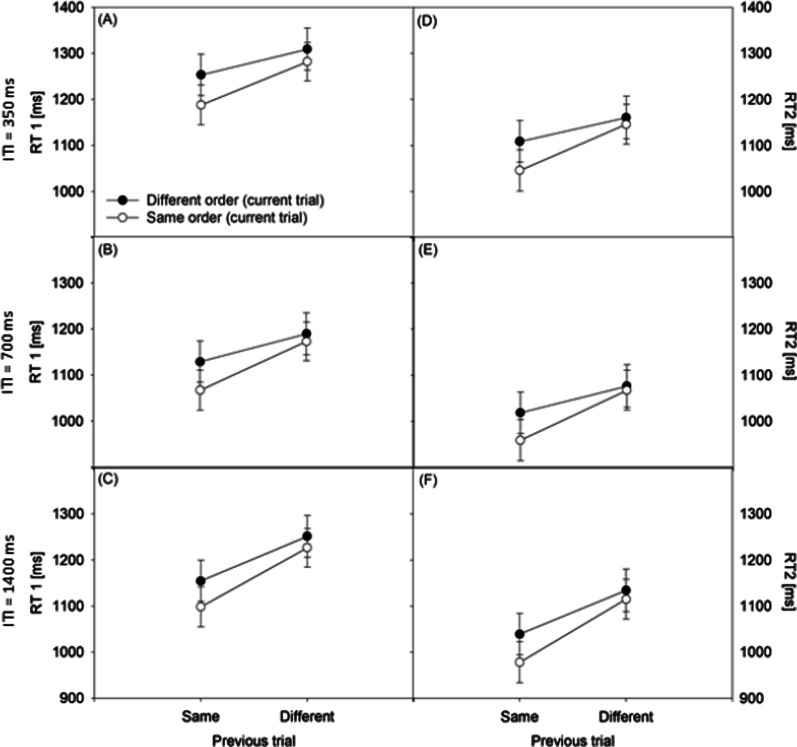
Reaction times of the first task (RT 1) and the second task (RT 2) in ms under current different-order and same-order conditions as well previous different-order and same-order conditions. Panel A ITI = 350 ms, RT1. Panel B ITI = 700 ms, RT1. Panel C ITI = 1,400 ms, RT1. Panel D ITI = 350 ms, RT2. Panel E ITI = 700 ms, RT2. Panel F ITI = 1,400 ms, RT2. *ITI* inter-trial interval

In the RT data, the main effect of CURRENT ORDER was significant, *F*(1, 87) = 163.335, *p* < 0.001, *ŋp*^2^ = 0.65. This effect generally shows order switch costs (e.g., [6, 16]); that is, RTs were shorter in same-order trials (*M* = 1,087 ms) than in different-order trials (*M* = 1,178 ms). In addition, the main effect of PREVIOUS ORDER was also significant, *F*(1, 87) = 69.248, *p* < 0.001, *ŋp*^2^ = 0.44, which demonstrates generally faster RTs in trial N when trial N − 1were same-order trials (*M* = 1,112 ms) in comparison to different-order trials (*M* = 1,152 ms).

The RT analysis also showed an interaction of CURRENT ORDER and PREVIOUS ORDER, *F*(1, 87) = 18.638, *p* < 0.001, *ŋp*^2^ = 0.18. Two sets of simple main effects basically replicated the findings of sequential modulations of order switch costs of Strobach et al. [1]. A first set of simple main effects showed that the order switch costs in trial N were significantly smaller after different-order trials (*M* = 70 ms), *F*(1, 87) = 83.803, *p* < 0.001, *ŋp*^2^ = 0.49, in comparison to after same-order trials (*M* = 112 ms), *F*(1, 87) = 136.935, *p* < 0.001, *ŋp*^2^ = 0.61. A second set of simple main effects demonstrated significantly shorter RTs in same-order trials N when trial N − 1were also same-order trials in comparison to different-order trials, *F*(1, 87) = 86.942, *p* < 0.001, *ŋp*^2^ = 0.50. Likewise, RTs were shorter in different-order trials N after same-order trials N − 1 than after different-order trials N − 1, *F*(1, 87) = 6.687, *p* < 0.05, *ŋp*^2^ = 0.07, although this difference was smaller, as reflected in the significant interaction of CURRENT ORDER and PREVIOUS ORDER.

The interaction of TASK, CURRENT ORDER and PREVIOUS ORDER demonstrated that the combined effects of CURRENT ORDER and PREVIOUS ORDER differed in RT1 and RT2, *F*(1, 87) = 11.602, *p* < 0.001, *ŋp*^2^ = 0.12. In same-order trials N, the increase from different-order trials N − 1 to same-order trials N − 1 was slightly higher in RT2, *F*(1, 87) = 138.106, *p* < 0.001, *ŋp*^2^ = 0.61, than in RT1, *F*(1, 87) = 131.372, *p* < 0.001, *ŋp*^2^ = 0.60. On the other hand, in different-order trials N, the increase from different-order trials N − 1 to same-order trials N − 1 was slightly higher in RT1, *F*(1, 87) = 88.463, *p* < 0.001, *ŋp*^2^ = 0.50, than in RT2, *F*(1, 87) = 77.887, *p* < 0.001, *ŋp*^2^ = 0.47. However, the combined effects of CURRENT ORDER and PREVIOUS ORDER were significant and basically similar in both, RT1 and RT2. In detail, the RT1 analysis also showed an interaction of CURRENT ORDER and PREVIOUS ORDER, *F*(1, 87) = 14.787, *p* < 0.001, *ŋp*^2^ = 0.15. A first set of simple main effects showed that the order switch costs in trial N were significantly smaller after different-order trials (*M* = 71 ms), *F*(1, 87) = 88.463, *p* < 0.001, *ŋp*^2^ = 0.50, in comparison to after same-order trials (*M* = 109 ms), *F*(1, 87) = 131.372, *p* < 0.001, *ŋp*^2^ = 0.60. A second set of simple main effects demonstrated significantly shorter RTs in same-order trials N when trial N − 1 were also same-order trials in comparison to different-order trials, *F*(1, 87) = 82.342, *p* < 0.001, *ŋp*^2^ = 0.49. Likewise, RTs were shorter in different-order trials N after same-order trials N − 1 than after different-order trials N − 1, although this difference was smaller, as reflected in the significant interaction of CURRENT ORDER and PREVIOUS ORDER, *F*(1, 87) = 10.170,, *p* < 0.001, *ŋp*^2^ = 0.11. The RT2 analysis showed an interaction of the factors CURRENT ORDER and PREVIOUS ORDER, *F*(1, 87) = 22.205, *p* < 0.001, *ŋp*^2^ = 0.20. A first set of simple main effects demonstrated that the task-order switch costs in trial N were significantly smaller after different-order trials (*M* = 68 ms), *F*(1, 87) = 77.887, *p* < 0.001, *ŋp*^2^ = 0.47, in comparison to after same-order trials (*M* = 138 ms), *F*(1, 87) = 138.106, *p* < 0.001, *ŋp*^2^ = 0.61. Note that the task-order switch costs and their reduction from same-order trial N − 1 to different-order trial N − 1 in RT2 could be due to a bottleneck mechanism and thus dependency between the processing in the first and the second task. This dependency might lead to forward propagation of the reduction of these costs in the first task to the reduction of these costs in the second task. This is indicated by highly significant correlations between equivalent reductions in the first and the second component tasks, *r*s > 0.91, *p*s < 0.001. (Importantly, assumptions of no bottleneck processing and dependency would theoretically assume no such correlation.) A second set of simple main effects revealed significantly shorter RT2s in same-order trial N when trial N − 1 were also same-order trials in comparison to different-order trials, *F*(1, 87) = 88.247, *p* < 0.001, *ŋp*^2^ = 0.50. There was no significant RT difference in the different-order trial N when trial N − 1 were same-order trials and different-order trials, *F*(1, 87) = 3.786, *p* > 0.06.

The interaction of CURRENT ORDER and ITI showed a non-significant trend, *F*(2, 87) = 2.774, *p* = 0.068, *ŋp*^2^ = 0.06, indicating slightly increasing task-order switch costs with increasing ITI of 350 ms (*M* = 75 ms) to 700 ms (*M* = 83 ms) and 1,400 ms (*M* = 114 ms). The main effect of TASK was significant, *F*(1, 87) = 149.006, *p* < 0.001, *ŋp*^2^ = 0.63, with shorter RT2 (*M* = 1,071 ms) in comparison to longer RT1 (*M* = 1,194 ms). This main effect was modulated by PREVIOUS ORDER, *F*(1, 87) = 9.843, *p* < 0.01, *ŋp*^2^ = 0.10, revealing slightly smaller task-order switch costs in trial N when trial N − 1 were same-order trials versus different-order trials for RT2 than for RT1. All interactions of the RT analysis including those with the factors ITI, PREVIOUS ORDER, and CURRENT ORDER were non-significant, *F*s < 1. All other main effects and interactions were also non-significant, *F*s < 1.007, *p*s > 0.36. Thus, there is no significant evidence supporting the modulation of the interaction of the task order of trial N − 1 and trial N by the interval between trials in the RT data.

Given the theoretical relevance of the null effect concerning the three-way interaction involving CURRENT ORDER, PREVIOUS ORDER, and ITI, we added a Bayesian analysis to evaluate the evidence for the lack of this interaction. More precisely, we conducted additional Bayesian Repeated Measures ANOVAs with default prior model probabilities for all models of 0.2 using JASP [17], separately for RT1 and RT2. To compute the Bayes factors for these three-way interactions, we added the main effects and the two-way interaction CURRENT ORDER X PREVIOUS ORDER to the null model. The Bayes factors BF_01_ for the models involving the three-way interaction of CURRENT ORDER, PREVIOUS ORDER, and ITI were 75.58 and 52.37, for RT1 and RT2, respectively.

### *Errors (Fig. **2**)*

**Fig. 2:**
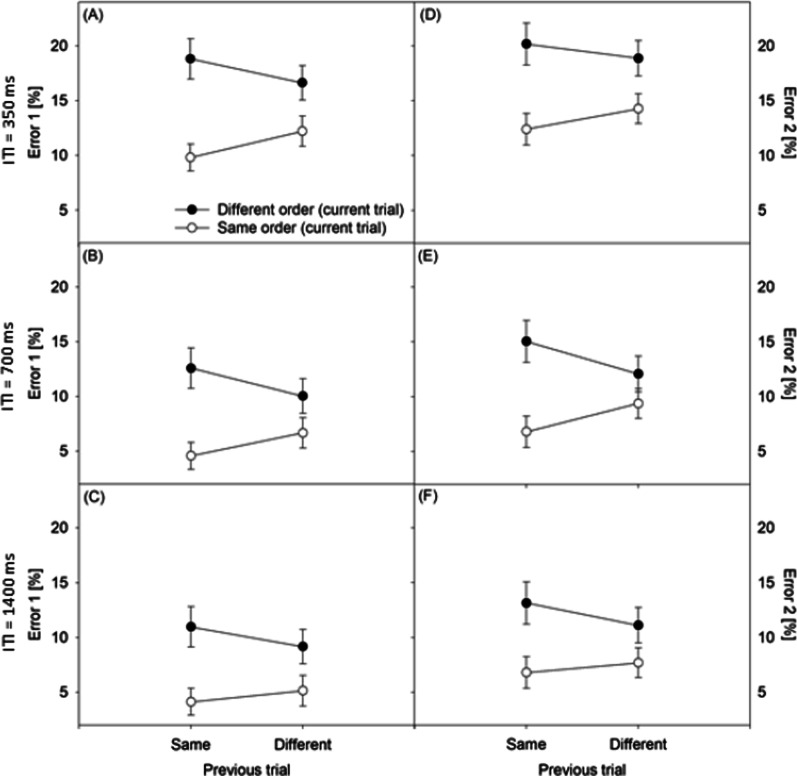
Error rates of the first task (Error 1) and the second task (Error 2) in percent (%) under current different-order and same-order conditions as well previous different-order and same-order conditions. Panel A ITI = 350 ms, RT1. Panel B ITI = 700 ms, RT1. Panel C ITI = 1,400 ms, RT1. Panel D ITI = 350 ms, RT2. Panel E ITI = 700 ms, RT2. Panel F ITI = 1,400 ms, RT2. *ITI* inter-trial interval

The analysis of the error rates showed a significant main effect of CURRENT ORDER, *F*(1, 87) = 136.054, *p* < 0.001, *ŋp*^2^ = 0.61, generally demonstrating order switch costs (e.g., [6, 16]). Error rates were lower in same-order trials (*M* = 8.3%) than in different-order trials (*M* = 14.1%). Importantly, the error rates revealed an interaction of the factors CURRENT ORDER and PREVIOUS ORDER, *F*(1, 87) = 62.815, *p* < 0.001, *ŋp*^2^ = 0.42. A first set of simple main effects demonstrated that the task-order switch costs in trial N were significantly smaller after different-order trials (*M* = 3.8%), *F*(1, 87) = 72.085, *p* < 0.001, *ŋp*^2^ = 0.45, than after same-order trials (*M* = 7.7%), *F*(1, 87) = 143.353, *p* < 0.001, *ŋp*^2^ = 0.62. A second set of simple main effects revealed lower rates in same-order trials N when trials N − 1 were also same-order trials in contrast to different-order trials, *F*(1, 87) = 30,349, *p* < 0.001, *ŋp*^2^ = 0.26. In comparison, error rates were higher in different-order trials N when the trials N − 1 were same-order trials than when they were different-order trials, *F*(1, 87) = 31.478, *p* < 0.001, *ŋp*^2^ = 0.27.

The effect of ITI on the error rates was significant, *F*(1, 87) = 6.471, *p* < 0.01, *ŋp*^2^ = 0.13. Error rates were higher under conditions of short ITIs (*M* = 15.4%) in comparison to the medium ITI condition (*M* = 9.7%) and the long ITI condition (*M* = 9.7%). The effect of Task was significant, *F*(1, 87) = 134.399, *p* < 0.001, *ŋp*^2^ = 0.61, demonstrating lower Error1 rates (*M* = 10.1%) than Error2 rates (*M* = 12.3%). With a non-significant trend, this main effect modulated CURRENT ORDER, *F*(1, 87) = 9.843, *p* < 0.01, *ŋp*^2^ = 0.10, revealing slightly smaller Error1 rate differences in comparison to Error2 rate differences in trial N. All interactions of the error rates analysis, including those with the factors ITI, PREVIOUS ORDER, and CURRENT ORDER, were non-significant, *F*s < 2.693, *p*s > 0.08. Thus, consistent to the RT data, there is no significant evidence supporting the modulation of the interaction of the task order of trial N − 1 and trial N by the interval between trials in the error data. Additional Bayesian Repeated Measures ANOVAs, analogous to the RT analyses, yielded Bayes factors BF_01_ of the models involving the three-way interaction of CURRENT ORDER, PREVIOUS ORDER, and ITI of 624.39 and 268.07, for Error1 rate and Error2 rate, respectively.

## Discussion

In the current study, we investigated task-order switch costs and their trial-to-trial modulation in dual tasks. We did so under conditions of three inter-trial intervals of different lengths to investigate the mechanisms underlying the modulation of these costs. Basically, our data replicated findings of task-order switch costs [1, 6, 8, 16], indicating impaired performance when two tasks are performed in different-order trials in comparison to same-order trials. Furthermore, we could replicate previous findings of sequential modulations of task-order switch costs [1]. That is, task-order switch costs were reduced after different-order trials N − 1 in comparison to after same-order trials N − 1.

Reminiscent of the findings of Strobach et al. [1] the sequential modulation of task-order switch costs took different forms in RTs and error rates. More precisely, whereas in error rates the reduction of the switch cost after different-order trials was characterized by decreased impairment in different-order trials N and decreased facilitation in same-order trials, RTs displayed a general increase after different-order trials. A straightforward explanation for this order-switch RT slowing is to assume the work of two different mechanisms, one leading to the reduction of the task-order switch costs and another one resulting in a general increase in response caution after order switch trials. As the latter mechanism should enhance RTs in both currently order repeat trials and order switch trials, one would expect, in the RT analysis, amplification versus masking of the effect of the first mechanism in order repeat trials and order switch trials, respectively. Similar two-process accounts have been applied to findings of „asymmetrical “ Gratton effects, that is, reduction of distractor congruency effects after trials associated with an incongruent distractor characterized by a selective post-incongruent increase in RTs of congruent trials in the absence of an effect in incongruent trials [18–20].

Most importantly, however, the three-way interaction involving CURRENT ORDER, PREVIOUS ORDER, and ITI did not approach statistical significance in any of our analyses. Furthermore, additional Bayesian analyses demonstrated considerably stronger evidence for a reference null model than for the model including the three-way interaction. Thus, our results lend no support to the notion that the sequential effect of task-order switch costs was modulated by the interval between trials. This temporal persistence appears incompatible with accounts assuming a short-lived, “phasic” set of task-order control, established by the order of previous task executions. According to these accounts we would expect a steady decay of the effect of the sequential modulation of task-order switch costs over time (i.e., with increasing ITI). It is also worth noting that the sequential modulation of task-order switch costs in RTs was more pronounced for Task 2 than for Task 1. In light of the fact that the presentation of the first stimulus of a trial unambiguously reveals the current trial’s task order, this finding demonstrates that disengagement from a disadvantageous task-order set (i.e., a low state of readiness for executing tasks presented in the order of the current trial) does not occur before the presentation of the second stimulus, that is, during the SOA of 400 ms.

### The current findings in the context of strategic expectancy-based accounts

Interestingly, task-order switch costs even seemed to increase alongside with the length of the inter-trial interval in both RT1 and RT2, although these effects were only marginally significant. Such an increase would clearly be consistent with accounts assuming that task-order switch costs are brought about by expectancy-based (time-consuming) preparation for the preceding task-order since implementation of the strategic set favoring trials associated with the expected task-order should need time to fully develop. Although assuming expectancy-based preparation does not predict the particular increase we observed—as at the current stage we cannot know how much time the effect takes to establish itself—neither passive persistence nor episodic retrieval would predict any increase at all. It is noteworthy in this connection, that the first account put forward to explain the Gratton effect (i.e., the reduction of the congruency effect in conflict tasks after an incongruent compared to after a congruent trial) assumed that participants expected a repetition of the congruency level of trial N − 1 [15]. Although, as laid out in the introduction, Egner et al. [13] observed a steady decrease of the Gratton effect with increasing inter-trial interval, an earlier study that compared the Gratton effect after a very short inter-trial interval (i.e., 50 ms) and after a longer inter-trial interval (of 200 ms) yielded the opposite result [21]. Neither of the studies may be considered conclusive regarding the time course of attentional adaptation, however, given various confounds of the sequence of congruency levels and both low-level and higher-level features (see [11], for an overview).

Nevertheless, the role of expectations in the domain of sequential effects in conflict tasks has become a prominent issue [22–24]. Of particular interest for discussing the results of our experiment, Erb and Aschenbrenner [25] suggested that multiple expectancies contribute to (modulations of) the Gratton effect. Specifically, these authors assumed that expecting both a repetition of the previous congruency level and repetition of the preceding sequence of congruency levels (i.e., repetition vs. switch) combine to yield a larger Gratton effect after repeating than after switching the congruency level in trial N − 1. In a similar vein, assuming that participants expect both a task-order repetition and a repetition of the previously experienced task-order sequence would, in principle, be consistent with the sequential modulations found in our study and in the study of Strobach et al. [1]. Table 2 presents an illustration of this multiple expectancies account.

**Table 2:** Correspondence ( +) and non-correspondence ( −) of expectancy for repetitions of the task-order and of the task-order sequence with current trials involving task order AB

Task-order sequence in trial N	Previous trial N − 2	Previous trial N − 1	Current trial N	Correspondence with previous task-order in trial N	Correspondence with previous task-order sequence in trial N
Same order → same order	AB	AB	AB	+	+
Different order → same order	BA	AB	AB	+	−
Different order → different order	AB	BA	AB	−	+
Same order → different order	BA	BA	AB	−	−

As can be seen in Table 2, the combined benefit of both expectations should be largest in same-order trials after same-order trials, whereas the combined cost should be maximal in different-order trials after same-order trials, and there would be intermediate effects in same-order trials after different-order trials and different-order trials after different-order trials. Assuming, in addition, a somewhat larger impact of expectancies for task-order repetition than for repetition of the task-order sequence would favor the former over the latter, thus predicting a pattern of results resembling the results obtained for error rates in the current study and by Strobach et al. [1].

### The current findings in the context of episodic bindings accounts

Rather than assuming multiple mechanisms (or multiple sources of a common preparatory mechanism), however, a simpler account of task-order priming might attribute both task-order switch costs and the sequential modulation thereof to a single process of facilitating trials whose task-order matches a previously experienced task-order (if it is assumed that the impact of previous experience is weighted by the recency of its occurrence). As mentioned in the introduction, episodic integration of tasks and temporal order of stimulus occurrence within a trial would constitute such an account. In this view, perceiving a task stimulus in the first or second temporal position of stimulus occurrence within a trial is associated with a tendency of retrieving previous episodes (more strongly for more recent episodes) containing the same stimulus (or stimulus category) or the same temporal position. Assuming episodic binding of the two pieces of information, mismatches of any of the two with trial N conditions would then impair response performance. Judging from studies investigating effects of episodic binding of basic visual features, such as stimulus shape and color, or simple motor responses, such effects are not subject to decay for periods of at least several seconds (e.g., [14]). That said, it should be noted that although binding of various basic stimulus and response features in memory episodes has been considered in extant models (e.g., [26, 27]) temporal order of events has to our knowledge not been among them hitherto. Moreover, a recent study by Moeller and Frings [28] failed to provide any evidence for such bindings involving simple key press responses, leading the authors to conclude that their results “provide unambiguous evidence that bindings do not include representations of the involved elements’ order, even if a sequence was clearly experienced “ (p. 330). As task-order priming based on episodic integration of tasks and temporal order would nicely account for the sequential modulation of task-order switch costs, however, more research on the issue of binding of temporal order information in various contexts including dual-task situations seems necessary.

## Methods

The methods applied in this study are similar to the methods of Group 1 of Strobach et al. [1]. Therefore, the present method section has overlapping passages with this study’s methods section.

### Participants

Participants were students of the Medical School Hamburg and other universities in the Hamburg area, recruited via online databases and personal contacts. All participants were German native-speakers and right-handed as investigated with the Edinburgh Handedness Inventory [29]; handedness is illustrated in form of the handedness laterality quotient where values between 1 (very low laterality) and 100 (full laterality) reflect right-handedness. They reported normal or corrected-to-normal hearing and vision and received course credit for their participation. All procedures performed in this study involving human participants were in accordance with the ethical standards of the institutional and/or national research committee and with the 1964 Helsinki declaration and its later amendments or comparable ethical standards. Informed consent was obtained from all participants before the commencement of the study. The study was approved by the ethical committee of Medical School Hamburg.

We included 30 participants into each experimental group (Group 1: 18 females, mean age: 22.7 years, age range: 18–28 years; mean handedness laterality quotient: 89.5, handedness laterality quotient range: 80–100; Group 2: 16 females, mean age: 23.3 years, age range: 20–30 years; mean handedness laterality quotient: 92.3, handedness laterality quotient range: 70–100; Group 3: 20 females, mean age: 25.4 years, age range: 21–30 years; mean handedness laterality quotient: 94.6, handedness laterality quotient range: 80–100). This sample size was based on an estimation of the required sample size using the G*Power software [30] for reliably testing the interaction of the factors CURRENT ORDER and PREVIOUS ORDER. We estimated an effect size of *ηp*^*2*^ = 0.15 based on the RT results of Strobach et al. [31]. The remaining parameters for the power analysis were specified as follows: Test family: F tests; Statistical test: ANOVA: Repeated measures, within-between interaction; Type of power analysis: a priori; α err prob: 0.05; Power (1 − β err prob): 0.8; Number of groups: 3; Number of measurements: 4; Corr. among rep measures: 0.00 (note that reducing the correlation between repeated measures to zero provides a rather conservative estimation of the required sample size, as increases in this correlation result in higher power and, thus, smaller required sample sizes); Nonsphericity correction ε: 1. This analysis yielded a required sample size of N = 78 in total across all groups. We increased this required sample size to 30 per group in order to compensate for potential dropout due to low accuracy or similar.

### Apparatus

Visual stimuli in the following experiments were presented on a 22-inch color monitor (Refresh rate: 60 Hertz; viewed from a distance of approximately 60 cm) and auditory stimuli were presented via headphones which were connected to IBM-compatible personal computers. Experiments were controlled by the experimental software package Presentation (Version 18).

### Stimuli

Participants performed a visual and an auditory choice RT task in the present dual-task situation. The auditory task included the presentation of sine-wave tones with pitches of either 350, or 1,650 Hz. Participants responded with the index (*M* key), and ring (*.* key) finger of the right hand, respectively; note that under conditions with 3 stimulus–response mappings instead of 2 stimulus–response mappings to manipulate task difficulty in Strobach et al. [1] a tone with medium frequency and middle-finger responses were added. The visual task included the presentation of small and large visually presented triangles and responses with the ring (*Y* key) and the index (*C* key) finger of the left hand, respectively; note that under conditions with 3 stimulus–response mappings instead of 2 stimulus–response mappings to manipulate task difficulty in Strobach et al. a triangle with a medium size and middle-finger responses were added.

### Procedure and design

Participants performed single-task blocks in which only 1 of the 2 tasks were presented. They also performed dual-task blocks that included the presentation of both tasks. Trials of single-task blocks started with the presentation of 3 dashes next to each other of which the middle dash was located at the centre of the screen. The dashes remained on the screen until the end of each trial while they disappeared between trials. An auditory stimulus (i.e., a sine-wave tone) appeared for 100 ms in auditory single-task block trials, or a visual stimulus (i.e., a triangle) appeared centrally in the visual single-task block trials 500 ms after onset of the presentation of the dashes and thus trial start; visual stimuli were presented until response or a maximum of 2,500 ms.

Similar to single-task trials, dual-task block trials started with the presentation of 3 white dashes that remained on screen until the end of each trial (or a maximum of 4,500 ms) and disappeared between trials. After 500 ms, a first stimulus (i.e., auditory or visual) was presented, followed by the presentation of the second stimulus (i.e., visual or auditory). The interval between the onsets of both stimuli (i.e., SOA) was 400 ms. Incorrect trials were completed with an error feedback (German word: “Fehler”) for 1,500 ms; incorrect trials included wrong or omitted responses as well as response reversals. Importantly, the ITI was varied between groups, so that Group 1, Group 2, and Group 3 performed trials with an ITI of 350 ms, 700 ms, and 1,400 ms, respectively. This way, the ITI was doubled from Group 1 to Group 2 and from Group 2 to Group 3.

Single-task blocks consisted of 32 single-task trials and stimuli were presented with equal frequency in a random order. In all 32 trials of the dual-task blocks, auditory and visual stimuli were presented with equal frequency and stimuli were selected randomly. The stimulus-order was random within blocks. Participants were instructed to respond as quickly and as accurately as possible in single-task blocks as well as in the dual-task blocks. In dual-task block trials, priority was instructed for the firstly presented stimulus.

Across the groups of this study, we conducted 2 different types of dual-task blocks with different task orders: dual-task blocks with random task order (*random dual tasks*) and dual-task blocks with fixed task order (*fixed dual tasks*). Dual-task trials with a first auditory stimulus (auditory-visual order trials) and with a first visual stimulus (visual-auditory order trials) were randomly mixed in random order dual-task blocks. Fixed order dual-task blocks included trials in a constant, fixed order. This order was verbally instructed to participants before the block start. These blocks exclusively included only trials with a first visual stimulus and a second auditory stimulus or these blocks exclusively included only trials with a first auditory stimulus and a second visual stimulus.

At the beginning of each session, one visual and 1 auditory single-task block was presented. Whereas half of the participants started with a visual block followed by an auditory block, the remaining participants performed the blocks in the opposite order. Following, two dual-task blocks with a fixed task order were conducted. Whereas half of the participants started with a dual-task block and trials with a first auditory stimulus followed by a block with trials with a first visual stimulus, the remaining participants performed the blocks in the opposite order. After this initial practice phase the *dual-task test* phase started including 20 random dual-task blocks. Each experimental session lasted approximately 60 min.

### Data analyses

Only trials from the dual-task test phase were analyzed. Before analyzing RTs and error rates, we excluded the first two trials of each random-order dual-task block. RTs (after the exclusion of errors on the choice decisions within the component tasks) and error rates (after the exclusion of errors on the choice decisions within the component tasks were aggregated into 4 conditions: (1) a same-order trial (trial N) following a same-order trial (trial N − 1), (2) a same-order trial (trial N) following a different-order trial (trial N − 1), (3) a different-order trial (trial N) following a same-order trial (trial), as well as (4) a different-order trial (trial N) following a different-order trial (trial N − 1); all participants included into the analysis had less than 30% excluded trials. These four conditions were analyzed in four-way mixed measures ANOVAs including the within-subjects factors CURRENT ORDER (same-order versus different-order trial in trial N), PREVIOUS ORDER (same-order versus different-order trial in trial N − 1), and Task (RT1 versus RT2/ Error 1 versus Error 2) as well as the between-subjects factor ITI (short ITI [Group 1], medium ITI [Group], and long ITI [Group 3]). This ANOVA was performed on RTs and error rates of Task 1 (firstly presented task, irrespective of either auditory or visual task) and Task 2 (secondly presented task, irrespective of either auditory or visual task): RT1 (RT of Task 1), RT2 (RT of Task 2), Error1 (errors in Task 1), and Error2 (errors in Task 2). Adjustment of task-order coordination would be generally indicated by an interaction of CURRENT ORDER and PREVIOUS ORDER. Therefore, we report the main effects and interactions with these factors first in the result sections. We also added Bayesian analyses to evaluate theoretically relevant null effects.

## Data Availability

The datasets used and/or analysed during the current study are available from the corresponding author on reasonable request.
